# Integrated Genomic Analysis Identifies ANKRD36 Gene as a Novel and Common Biomarker of Disease Progression in Chronic Myeloid Leukemia

**DOI:** 10.3390/biology10111182

**Published:** 2021-11-15

**Authors:** Zafar Iqbal, Muhammad Absar, Tanveer Akhtar, Aamer Aleem, Abid Jameel, Sulman Basit, Anhar Ullah, Sibtain Afzal, Khushnooda Ramzan, Mahmood Rasool, Sajjad Karim, Zeenat Mirza, Mudassar Iqbal, Maryam AlMajed, Buthinah AlShehab, Sarah AlMukhaylid, Nouf AlMutairi, Nawaf Al-anazi, Muhammad Farooq Sabar, Muhammad Arshad, Muhammad Asif, Masood Shammas, Amer Mahmood

**Affiliations:** 1 Department of Clinical Laboratory Sciences (CLAB), College of Applied Medical Sciences (CoAMS-A), King Saud Bin Abdulaziz University for Health Sciences (KSAU-HS), King Abdulaziz Medical City (KAMC)/Kind Abdullah International Medical Research Centre (KAIMRC)/Saudi Society for Blood and Marrow Transplantation (SSBMT), National Guard Health Affairs (NGHA), Al-Ahsa 31982, Saudi Arabia; almajed018@ksau-hs.edu.sa (M.A.); alshehab019@ksau-hs.edu.sa (B.A.); almukhaylid034@ksau-hs.edu.sa (S.A.); almutairin010@ksau-hs.edu.sa (N.A.); anaziNM@ngha.med.sa (N.A.-a.); 2 Hematology, Oncology and Pharmaco-genetic Engineering Sciences (HOPES) Group, Health Sciences Laboratories (HaSiL), Department of Zoology, University of the Punjab (ZPU), Lahore 54590, Pakistan; mashwani82@gmail.com (M.A.); dr_tanveerakhtar@yahoo.com (T.A.); 3 Next-Generation Medical Biotechnology & Genomic Medicine Division, Department of Biotechnology, Qarshi University, Lahore 54000, Pakistan; 4 Division of Hematology/Oncology, Department of Medicine, King Khalid University Hospital (KKUH), College of Medicine, King Saud University, Riyadh 14800, Saudi Arabia; aameraleem@hotmail.com; 5 Hayatabad Medical Complex, Peshawar 25100, Pakistan; ajameel99@yahoo.com; 6 Center for Genetics and Inherited Diseases, Taibah University Madinah, Medina 30001, Saudi Arabia; sbasit.phd@gmail.com; 7 National Heart and Lung Institute, Imperial College London, London SW3 6LY, UK; anharullah@gmail.com; 8 Biomedical Research Laboratory, College of Medicine, Alfaisal University, Riyadh 11533, Saudi Arabia; safzal@alfaisal.edu; 9 Research Centre, King Faisal Specialist Hospital and Research Centre, Riyadh 11533, Saudi Arabia; khushnooda@gmail.com; 10 Center of Excellence in Genomic Medicine Research (CEGMR), Department of Medical Laboratory Technology, Faculty of Applied Medical Sciences, King Abdulaziz University, Jeddah 21589, Saudi Arabia; mahmoodrasool@yahoo.com (M.R.); sajjad_k_2000@yahoo.com (S.K.); 11 King Fahd Medical Research Center, Department of Medical Laboratory Technology, Faculty of Applied Medical Sciences, King Abdulaziz University, Jeddah 21589, Saudi Arabia; zmirza1@kau.edu.sa; 12 Asian Medical Institute, Kant 725012, Kyrgyzstan; drmudassar440@gmail.com; 13 Division of Hematology/Oncology, Department of Pediatrics, King Abdulaziz Hospital, Al-Ahsa 36428, Saudi Arabia; 14 Centre for Applied Molecular Biology (CAMB), University of the Punjab, Lahore 54590, Pakistan; farooqsabar@yahoo.com; 15 Department of Biological Sciences, International Islamic University, Islamabad 44000, Pakistan; arshimalikk@gmail.com; 16 Research Operations and Development, BUITEMS, Quetta 87300, Balochistan, Pakistan; asifjallali@yahoo.com; 17 Department of Medical Oncology, Harvard (Dana Farber) Cancer Institute, Boston, MA 02115, USA; masood_shammas@dfci.harvard.edu; 18 Cancer Stem Cell Unit, Department of Anatomy, King Saud University, 11461 Riyadh, Saudi Arabia; amer_dk@yahoo.com

**Keywords:** CML, disease progression, common biomarker, drug target, ANRD36

## Abstract

**Simple Summary:**

Chronic myeloid leukemia is a type of blood cancer that is regarded as a success story in determining the exact biological origin, pathogenesis and development of a molecularly targeted (mutation-specific) therapy that has led to successful treatment of this fatal cancer. It is caused by the BCR-ABL fusion gene, which is formed from the translocation between chromosomes 9 and 22. Anti-BCR-ABL drugs, known as tyrosine kinase inhibitors (TKIs), have led to long-term remissions in more than 80% of CML patients and even cure in about one-third of patients. Nevertheless, many patients face drug resistance, and disease progression occurs in about 30% of CML patients, leading to morbidities and mortality. Unfortunately, no biomarkers of CML progression are available due to a poor understanding of the mechanism of progression. Therefore, finding reliable molecular biomarkers of CML progression is one of the most attractive research areas in 21st-century cancer research. In this study, we report novel genomic variants exclusively found in all our advanced-phase CML patients. This study will help in identifying CML patients at risk of disease progression and timely therapeutic interventions to avoid or at least delay fatal disease progression in this cancer.

**Abstract:**

**Background:** Chronic myeloid leukemia (CML) is initiated in bone marrow due to chromosomal translocation t(9;22) leading to fusion oncogene BCR-ABL. Targeting BCR-ABL by tyrosine kinase inhibitors (TKIs) has changed fatal CML into an almost curable disease. Despite that, TKIs lose their effectiveness due to disease progression. Unfortunately, the mechanism of CML progression is poorly understood and common biomarkers for CML progression are unavailable. This study was conducted to find novel biomarkers of CML progression by employing whole-exome sequencing (WES). **Materials and Methods**: WES of accelerated phase (AP) and blast crisis (BC) CML patients was carried out, with chronic-phase CML (CP-CML) patients as control. After DNA library preparation and exome enrichment, clustering and sequencing were carried out using Illumina platforms. Statistical analysis was carried out using SAS/STAT software version 9.4, and R package was employed to find mutations shared exclusively by all AP-/BC-CML patients. Confirmation of mutations was carried out using Sanger sequencing and protein structure modeling using I-TASSER followed by mutant generation and visualization using PyMOL. **Results**: Three novel genes (ANKRD36, ANKRD36B and PRSS3) were mutated exclusively in all AP-/BC-CML patients. Only ANKRD36 gene mutations (c.1183_1184 delGC and c.1187_1185 dupTT) were confirmed by Sanger sequencing. Protein modeling studies showed that mutations induce structural changes in ANKRD36 protein. **Conclusions**: Our studies show that ANKRD36 is a potential common biomarker and drug target of early CML progression. ANKRD36 is yet uncharacterized in humans. It has the highest expression in bone marrow, specifically myeloid cells. We recommend carrying out further studies to explore the role of ANKRD36 in the biology and progression of CML.

## 1. Introduction

Chronic myeloid leukemia (CML) is a neoplasm of hematopoietic cells, which is characterized by a deregulated high production of immature granulocytes and their progenitors [1]. Since these cells are immature, they are not fully functional [2]. The excessive proliferation of progenitor cells and blasts results in a change in the balance between regeneration and differentiation [3]. Approximately 15% of all leukemias are CML, which means that 2 out of 100,000 individuals develop CML yearly. Out of those patients, 5–10% have exposure to excessive radiation [4].

CML was the first neoplasm to be linked to a chromosomal abnormality, and it is also one of the most intensely investigated malignancies [3]. CML is instigated by a reciprocal chromosomal translocation t(9;22) giving rise to Philadelphia chromosome [5]. The translocation ensues between ABL proto-oncogene on the long arm of chromosome 9 and breakpoint cluster region (BCR) on chromosome 22, giving rise to BCR-ABL fusion oncogene [6]. This fusion oncogene encodes a new oncoprotein called bcr-abl [7,8]. The bcr-abl oncoprotein has enhanced tyrosine kinase activity that hinders apoptosis, alters cell cycles and deregulates cell division, leading to leukemogenesis [9,10]. In the last two decades, tyrosine kinase inhibitors (TKIs) have revolutionized CML treatment; recently, the overall survival of CML patients has been brought equal to that of the general public due to the introduction of TKIs [11].

There are three main disease phases of CML, namely chronic phase (CP), accelerated phase (AP) and blast-crisis phase (BC) [12]. Most of the CML patients are diagnosed in the chronic phase, and hence overall survival of CML is excellent [13]. Nevertheless, about 20% of CML patients progress to advanced phases of the disease that result in drug resistance, intolerance, morbidities and mortality [14]. Unfortunately, the mechanism of CML progression is poorly comprehended [15]. Moreover, universal biomarkers for early diagnosis of disease progression are not available. The discovery of common biomarkers for CML progression can help in the early determination of CML patients at risk of progression and the clinical management of these patients to avoid or delay disease progression [16,17]. Therefore, this study was intended to determine common gene variants associated with CML progression using high-throughput DNA sequencing methods such as whole-exome sequencing.

## 2. Materials and Methods

### 2.1. Patient Inclusion and Exclusion Criteria

The study was carried out from January 2012 until December 2019. One hundred forty-one CML patients were enrolled in the study from Hayatabad Medical Complex (HMC) Peshawar, Khyber Pakhtunkhawa (KP), Pakistan. Peripheral blood samples were collected from all CML patients along with clinical data. Out of 141 patients, 123 were CP-CML, 12 were AP-CML and 6 were BC-CML. AP- and BC-CML patients were the experimental group, while CP-CML patients were included as controls. Additionally, 10 age/gender-matched healthy controls were included in the study.

Regarding treatment, imatinib mesylate (IM) was the first line of therapy for all patients. However, nilotinib (NI) was prescribed in case of IM resistance. The criteria of all responses were per European LeukemiaNet guidelines 2013 [18].

### 2.2. Definitions of Clinical Phases of Chronic Myeloid Leukemia (CML) for Staging

Chronic phase (CP) was identified by the presence of three main parameters in the circulation, which are 15–19% basophils, less than 30% blasts and promyelocytes and less than 5% blast cells. Moreover, evidence of blast cells in extramedullary sites was not available [19]. Accelerated phase (AP) was described by an increase of blasts up to 15–29%, or 30% promyelocytes in bone marrow or blood. Furthermore, ≥20% basophils and constant low platelet counts of less than 100 × 10^9^/L were detected, and chromosomal abnormalities in Philadelphia cells were discovered [20]. Blast crisis (BC) was defined by the presence of blasts equal to or greater than 30% in bone marrow or blood. In BC, blasts were present in the spleen and in other extramedullary sites [18].

### 2.3. Criteria for Assessment of Treatment Response in Chronic Myeloid Leukemia

Patient blood count and physical examination were performed every 4–8 weeks to monitor treatment response. The listed response tools were applied to evaluate the effectiveness of CML medication in all patients [21,22].

#### 2.3.1. Complete Hematological Response (CHR)

CHR was defined as the absence of immature cells, normal platelet count of less than 450 × 10^9^/L and normal basophil count of less than 5%. Impalpable spleen was also documented [18,23].

#### 2.3.2. Cytogenetic Response (CyR)

Cytogenetics and differential morphology or FISH bone marrow aspirates were evaluated for diagnosis every 6 and 12 months. Cytogenetic testing results of Ph+: complete cytogenetic response (CCyR): 0% or less than 1% BCR-ABL nuclei by FISH/≥200 cells, partial (PCyR): Ph+ = 1–35%, minor cytogenetic response (mCyR): Ph+ = 36–65%, minimal cytogenetic response (miCyR): Ph+ = 66–95%, no cytogenetic response (nCyR): Ph+ > 95% [18,22,23].

#### 2.3.3. Criteria for Calculation of Molecular Response (MR)

Major molecular response (MMR) was described as a BCR-ABL/ABL ratio cut-off of ≤0.1%. Moreover, a ratio of ≤0.0032% was termed as MR [4,5,18,22,23].

### 2.4. Criteria for Calculation of European LeukemiaNet (ELN) Responses and Survival

The following criteria were used for calculating European LeukemiaNet (ELN) treatment responses and survivals:

**Optimal response**: It was defined at 3 months if Ph+ = ≤ 35%, at 6 months if Ph+ = 0, at 12 months if BCR-ABL1 by PCR was ≤0.1% then and at any time if BCR-ABL1 ≤ 0.1% [18].

**Warning**: It was defined at baseline as high risk or CCA/Ph+ major route, at 3 months Ph+ 36–95%, at 6 months Ph+ 1–35% and at 12 months if BCR-ABL1 by PCR = > 0.1–1% [18].

**Failure**: It was defined at 3 months as non-CHR and/or Ph+ > 95%, at 6 months as Ph+ > 35%, at 12 months as Ph+ > 0 and then at any time as loss of CHR or loss of CCyR [18].

**Overall Survival (OS)**: The overall survival was taken as the beginning of the IM therapy to the patient expired date or last follow-up [24].

**Progression-Free Survival (PFS)**: PFS was measured from the day IM began to the development of CML to AP or BC or to death. Any patient who survived as of the last day of study was censored at the last follow-up date. The confirmation of the survival status of patients who were absent from the last follow-up was conducted by contacting patients based on the registered contact information. The survival analysis was determined as per Kaplan–Meier method [25].

### 2.5. Criteria for Documenting Adverse Events

According to the standard terminologies (version 4.03), hematological undesirable effects were categorized [19].

### 2.6. Ethical Approval

The protocols of this study were approved by King Abdullah International Medical Research Center (KAIMRC); King Saud bin Abdulaziz University for Health Sciences (KSAU-HS), Hayatabad Medical Complex (HMC), Peshawar, Pakistan; and University of the Punjab, Lahore, Pakistan. Written informed consent was obtained from every enrolled patient in this study. The study was carried out per regulations of the Declaration of Helsinki [26,27].

### 2.7. Sample Collection and DNA Extraction

Ten milliliters of peripheral blood was collected in EDTA tubes (BD Vacutainer Systems, Franklin Lakes, NJ, USA). QIAamp DNA Blood Mini Kit (QIAGEN) was used to extract DNA from all patients [28]. DNA quantitation was performed by utilizing NanoDrop Spectrophotometer (NanoDrop Technologies, Inc., Wilmington, DE, USA). After that, DNA was diluted into aliquots of 70–80 ng/μL for mutation detection by whole-exome sequencing (WES). The excess amount of DNA was diluted to 40 ng/μL for Sanger sequencing. DNA was stored in a freezer at −80 °C [29].

### 2.8. Whole-Exome Sequencing

In this study, the SureSelect^XT^ V6-Post Capture Exome kit (Agilent Technologies Inc., Santa Rosa, CA, USA) was utilized for the formulation of libraries and target enrichment. For exonic and intron flanking regions, exome enrichment was done by SureSelect^XT2^ Target Enrichment System for Illumina Paired-End Multiplexed Sequencing (Illumina, San Diego, CA, USA) based on the manufacturer protocol (Agilent Technologies Inc., Santa Rosa, CA, USA). DNA fragmentation and tagmentation were performed per manufacturer’s protocols. Following that, purification and amplification of the DNA were conducted. Magnetic beads were used to purify the amplified DNA fragments. The whole exome was used to capture target regions. Subsequently, PCR amplified the enriched DNA fragments. To enumerate the augmented fragments, the Qubit fluorometer was operated on the enriched libraries. Moreover, using Agilent Bioanalyzer (Agilent Technologies Inc., Santa Rosa, CA, USA), the library size distribution was quantified. Last of all, for cluster generation and whole-exome sequencing, the amplified DNA fragments were loaded on a flow cell on an Illumina NextSeq500 instrument (Illumina, San Diego, CA, USA) [30].

### 2.9. Exome Sequencing Data Analysis

The WES output BCL records were transformed to FASTQ files with the aid of BCL2FASTQ software. The BWA-MEM algorithm aided in the alignment of the FASTQ records to the human genome (GRCh37/hg19), using the BWA aligner. Whole-exome sequencing data statistics including sequencing depth and some summary statistics are presented in Table 1 and Table 2. For variant analysis, the Genome Analysis Toolkit (GATK) was utilized. Illumina Variant Studio was used for genomic variant annotation and filtration [31]. The resulting annotated files on average had approximately 90,000 variants. This includes synonymous, coding, intergenic, intronic, splice-site and 5′ and 3′ UTR variants. Variant statistics are presented in Table 3.

### 2.10. Primary Analysis

In order to identify a shared biomarker for CML growth, mutated genes were analyzed in all advanced-phase CML patients. An Excel file presenting the WES was modified using the filtration strategy, which excluded all synonymous and intron variants while rare variants were called. Moreover, all recognized tolerant (T) and benign (B) variants (with known prediction) were eliminated. For multiple B or T, we considered it B if the frequency of B was ≥70%. On the other hand, it was thought to be T if the frequency of T was ≥70% [32]. In summary, synonymous, intergenic and deep intronic variants were removed from the annotated file. Only those variants having high and intermediate protein effects and splice variants were retained. Moreover, variants with a population frequency of more than 0.005 in the dbSNP and Exome Sequencing Project (ESP) database were also removed. A total of approximately 124 on average rare variants were obtained as a result of this analysis. Finally, further data analysis was performed to find driver mutations in novel genes, i.e., mutations that are shared by all advanced-phase CML patients but absent in chronic-phase CML or healthy controls. Hence, these variants might have a significant role in disease progression [16,17]. Data generated from next-generation sequencing have been submitted to NCBI and can be accessed at https://www.ncbi.nlm.nih.gov/sra/PRJNA734750 (SRA accession number PRJNA734750; accessed on 7 August 2021).

### 2.11. Validation of Mutation by Sanger Sequencing

In order to validate the WES detected variants, Sanger sequencing was carried out in all samples under investigation. For Sanger sequencing, primers for different genes under investigation were designed using Primer 3 software. Primers were ordered from Applied Biosystems, California, CA, USA. Target amplicons in the genes were amplified using PCR. DNA sequencing reactions were prepared using ABI PRISM Big Dye Terminator Cycle Sequencing Ready Reaction kits (Applied Biosystems, California, CA, USA) [33]. Then, forward and reverse DNA templates were sequenced by Sanger sequencing using ABI Prism 3730 Genetic Analyzer (Applied Biosystems, California, CA, USA) [34,35].

### 2.12. Statistical Analysis of Patient Clinical Data

Based on the normality test, absolute numbers and percentages were demonstrated for categorical variables; mean and an appropriate measure of variation were demonstrated for continuous variables. For categorical data, chi-square or Fisher’s exact test were used to compare two groups, while a two-sample independent test or Mann–Whitney U test was used for the continuous data. ANOVA or Kruskal–Wallis test was used to analyze variance for groups of ≥3. To assess the survival outcome, Kaplan–Meier survival analysis curves were plotted [25]. The group comparison was performed by log-rank test. SAS/STAT software version 9.4 was used for data analysis (SAS Institute Inc., Cary, NC, USA). For statistical computing, the R package was employed (Vienna, Austria) [36]. The Eutos risk score, Euro risk score and Sokal risk score were measured [18,37,38,39,40].

### 2.13. Protein Modeling Studies

One of the most significant issues in computational structural biology is the prediction of 3-dimensional protein structures from amino acid sequences. The protein structure of ANKRD36, which is yet uncharacterized in humans, was modeled using I-TASSER webserver [41]. It resulted in computational prediction of its structure and an assessment of these mutations [41]. Mutagenesis was specifically done on residues 395 and 396 using PyMOL Wizard. Further, the wild-type and mutated structures were superimposed using Schrodinger’s PyMOL Molecular Graphics System, Version 2.5 [42].

## 3. Results

A total of 141 CML patients were included in this study. Mean age of the patients was 34.6 years (Table 3), and male-to-female ratio was 1.6:1. Gender statistics revealed that females were 60.2% and males were 39.8%. The mean hemoglobin was 10.1, and the mean WBC count was 317.9. In addition, the platelet count in CML patients was 400.2. Overall, females were more commonly affected by CML.

During course of study, 12.8% (*n* = 18) of patients progressed to advanced phases (AP = 2, BC = 12). CP-, AP- and BC-CML patients had mean ages of 33.5, 35.6 and 38.1 years, respectively. In addition, there was male dominance found in all the CML phases. Furthermore, the male-to-female ratio was calculated to be 2:1, 2:1 and 1.5:1 in BC, AP and CP, respectively. Moreover, anemia was common among two-thirds of the patients. Of all CML patients, 56% of them had a leukocyte count 50 × 10^9^/L or higher (*n* = 79). Imatinib was first-line TKI, and it was administered to 66.7%, 66.7% and 58.36% of CP, AP and BC CML patients, respectively. Chemotherapy was given to 8.1%, 66.7% and 75% of CP, AP and BC CML patients, respectively. Overall, 12.7% of CML patients (*n* = 18) developed to AP-CML (*n* = 6) or progressed to BC-CML (*n* = 12) (Table 4).

There was a significant difference between chronic- and advanced-phase patients with respect to male-to-female ratio, hemoglobin level, WBC count, platelet count, type of treatment received, hepatomegaly, splenomegaly and survival status (Table 4 and Table 5).

**Table 3:** Comparisons between our findings and other studies.

Characteristics	Japan	Iraq [43]	US [44]	EU [45]	India [46]	Our Study
# of Patients	506	100	1106	210	90	141
Mean Age, years		51.7	41.1	55	38.6	36.4
Male	349	58%	59%	54%	57%	60.2%
Female	157	42%	41%	46%	42.2%	39.8%
Male:Female Ratio	2.2:1	1.4:1	1.4:1	1:1	1.4:1	1.6:1
Hemoglobin (g/dL) Mean	4	12.28		12.6	9.41	10.1
WBC count (×10^9^/L) Mean		45.26	19	80.2	182	317.9
Platelets (×10^9^/L) Mean	47.2	341.5	77	373	328	400.2

**Table 4:** Comparison of demographics, clinical data and laboratory parameters between three phases of CML.

Characteristics	Patient Group			
	CP-CML, n (%)	AP-CML, n (%)	BC-CML, n (%)	*p*-Value
# of Patients	123 (87.2)	6 (4.3)	12 (8.5)	
Age, Years				
Mean (Range)	35.5 (9–7)	35.6 (27–43)	38.1 (29–50)	
Gender				
Male	74 (60.2)	4 (66.67)	8 (66.7)	*p* = 0.6004
Female	49 (39.8)	2 (33.33)	4 (33.3)	*p* = 0.5987
*p*-Value	*p* = 0.0272	*p* = 0.3980	*p* = 0.2933	
Male:Female Ratio	1.5:1	2:1	2:1	
Hemoglobin (g/dL) Mean	10.1			
<12g/dl	69 (56.1)	5 (83.3)	9 (75)	*p* = 0.0642
>12g/dl	14 (11.4)	1 (16.7)	3 (25)	*p* = 0.2609
*p*-Value	*p* = 0.0024	*p* = 0.2154	*p* = 0.1380	
WBC count (×10^9^/L) Mean	313.7	315	325	
<50	20 (16.3)	1 (20)	2 (16.7)	*p* = 0.8276
>/=50	64 (52)	5 (80)	10 (83.3)	*p* = 0.0184
*p*-Value	*p* = 0.0052	*p* = 0.2752	*p* = 0.0661	
Platelets (×10^9^/L) Mean	400.2			
<450	75 (61)	4 (66.7)	10 (83.3)	*p* = 0.2528
>/=450	33 (26.8)	2 (33.3)	2 (16.7)	*p* = 0.8722
*p*-Value	*p* = 0.0011	*p* = 0.4786	*p* = 0.0661	
Imatinib				
Yes	82 (66.7)	4 (66.7)	7 (58.3)	*p* = 0.7260
Nilotinib as 2nd Line				
Yes	41 (33.3)	4 (66.7)	8 (66.7)	*p* = 0.0065
Hydroxyurea				
Yes	82 (66.7)	3 (50)	10 (83.3)	*p* = 0.9967
Interferon				
Yes	41 (33.3)	0 (0)	0 (0)	*p* = 0.0038
Chemotherapy				
Yes	10 (8.1)	4 (66.7)	9 (75)	*p* < 0.0001
Splenomegaly				
<5 cm	4 (3.3)	0 (0)	0 (0)	*p* = 0.4358
5–8 cm	9 (7.3)	1 (16.7)	3 (25)	*p* = 0.0619
>8 cm	70 (56.9)	5 (83.3)	9 (75)	*p* = 0.0732
No splenomegaly	40 (32.5)	0 (0)	0	*p* = 0.0044
Hepatomegaly				
Yes	35 (28.5)	4 (66.7)	8 (66.7)	*p* = 0.0014
Anemia				
Yes	97 (78.9)	5 (83.3)	9 (75)	*p* = 0.9807
Pregnant				
Yes	4 (8.2)	0 (0)	0 (0)	*p* = 0.2090
Survival Status				
Confirmed Deaths	10 (8.1)	0 (0)	9 (75)	*p* = 0.0003
Alive at Last Follow-Up (Overall Survival)	113 (91.9)	6 (100)	3 (25)	*p* = 0.0003

**Table legend**: WES: whole-exome sequencing; WBC: white blood cell; CP: chronic phase; AP: accelerated phase; BC: blast phase; CP-CML: chronic-phase chronic myeloid leukemia; AP-CML: accelerated-phase chronic myeloid leukemia; BC-CML: blast-phase chronic myeloid leukemia.

**Table 5:** Different types of variants identified in each exome sequenced sample.

Variant Type	ID 1	ID 2	ID 3	ID 4	TD 5
Number of SNPs	88,892	90,562	88,725	90,441	86,484
Synonymous Variants	11,945	12,268	11,810	12,053	11,444
Missense Variants	11,139	11,467	11,116	11,408	10,776
Stop Gained	88	111	107	109	107
Stop Lost	41	40	48	44	41
Number of INDELs	9911	10,000	10,126	10,003	9637
Frameshift Variants	312	310	322	322	296
Inframe Insertions	178	169	165	175	166
Inframe Deletions	200	195	208	186	184
% found in dbSNP142	97.1	97.0	96.9	96.9	96.9
Het/Hom Ratio	1.4	1.7	1.3	1.6	1.1
Ts/Tv Ratio	2.3	2.3	2.3	2.3	2.3

Het/Hom ratio: ratio of number of heterozygous variants to number of homozygous variants; Ts/Tv ratio: ratio of transition rate of SNVs that pass the quality filters divided by transversion rate of SNVs that pass the quality filters.

### 3.1. Exome Sequencing: Initial Screening for Novel Genes

Rare variants, as well as the variants that were absent in the population variation databases, were prioritized for further analysis. Initially, 55 candidate variants in 22 genes were prioritized based on filtration criteria described in Section 2. Statistics of variants are provided in Table 5. Variants in advanced-phase CML patients were filtered. Three novel genes (ANKRD36, ANKRD36B and PRSS3) were found mutated in all advanced-phase CML patients but not in CP-CML and healthy controls. Data generated from next-generation sequencing have been submitted to NCBI and can be accessed through at https://www.ncbi.nlm.nih.gov/sra/PRJNA734750 (SRA accession number PRJNA734750; accessed on 7 August 2021).

### 3.2. Mutation Validation by Sanger Sequencing

**Figure 1:**
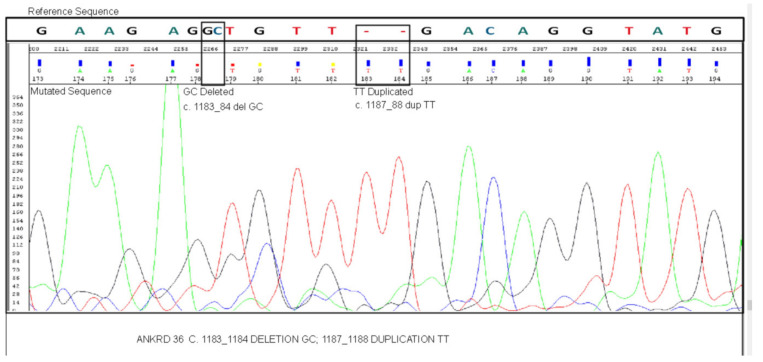
Confirmation of the presence of c.1183_1184 delGC and c.1187_1188 dupTT mutation in ANKRD36 gene by Sanger sequencing in unknown gene variants common in accelerate/blast phase (AP/BC) CML patients (AP, *n* = 5; BC, *n* = 7).

ANKRD36B (c.2758A > G) and PRSS3 (c.473_474insCC and c.478_479delAC) variants were not confirmed using Sanger sequencing. However, ANKRD36 gene mutations (c.1183_1184 delGC and c.1187_1188 dupTT) were confirmed by Sanger sequencing in BC- CML patients (Figure 1), demonstrating the association between ANKRD36 variants and CML progression. ANKRD36 mutations were confirmed in AP-CML as well, showing that these mutations are an early indicator of CML progression. This also shows that ANKRD36 mutations are a potential early biomarker of CML progression.

### 3.3. Protein Modeling Studies

The structure of the protein encoded by ANKRD36 was unknown and no prior PDB deposit was available. Therefore, ANKRD36 modeling studies were carried out using ANKRD36 protein sequence retrieved from UniProt [47] (https://www.uniprot.org/uniprot/A6QL64; accessed on 7 August 2021). Computational prediction of the protein structure was done using the I-TASSER webserver. The mutation was manually evaluated, and the wild and mutated structures were superimposed using PyMOL to shed light on structural changes induced.

**Figure 2:**
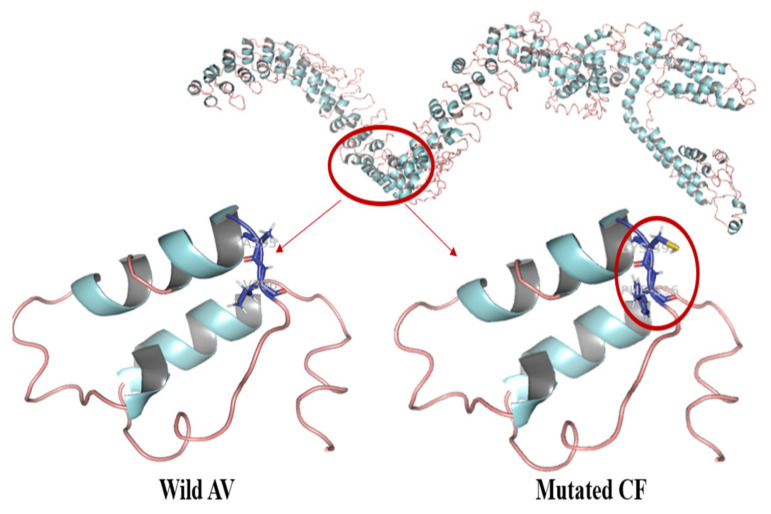
Protein modeling studies of normal wild-type and mutated ANKRD36.

The effect of nonsynonymous missense mutations is shown in Figure 2, wherein we zoomed into the region harboring the two nonsynonymous missense mutations.

Our analysis shows that these mutations induced structural changes in ANKRD36 protein due to the incorporation of bigger cysteine (Cys) and phenylalanine (Phe) residues instead of the comparatively smaller alanine (Ala) and valine (Val) on residues 395 and 396, respectively (Figure 2). The RMSD was in range of 0.025–0.043 [47,48]. A395C mutation has not been previously reported and could be of significance. Functional changes and possible pathogenesis associated with ANKRD36 gene may have been due to these mutations that lead to structural changes in the protein encoded by ANKRD36. This analysis also indicates that mutated ANKRD36 protein may have an important role in CML progression and may be a potential new drug target in CML progression.

## 4. Discussion

This study included overall 141 patients from different phases of CML. In our study, mean age of the patients was 36.4 ± 5.2 years. It is important to mention that the mean age of our CML patients is significantly different from that of Western populations. In Europe, the mean age of CML patients was 55 years [45]. A study reported the mean age of CML patients in the USA to be 41.1 ± 13.3 years [44]. In Japan, the mean age of patients diagnosed with CML was 56 years [49]. Due to this factor, the life expectancy of CML patients is not comparable to the general population in developing countries as it is for developed countries such as the United States, Europe and Japan [44,45,49]. Furthermore, there was a significant difference between chronic- and advanced-phase patients with respect to male-to-female ratio, hemoglobin level, WBC count, platelet count, splenomegaly and survival status, which is in accordance with previous reports [44,45,49].

During course of study, 12.8% (*n* = 18) of patients progressed to advanced phases (AP = 6, BC = 12). A European study reported 9 (4.2%) out 210 enrolled CML patients developed to advanced phases (AP = 5, BC = 4) [50]. Based on the results of the pivotal International Randomized Study of Interferon and STI571 (IRIS) trial involving 1106 randomized patients newly diagnosed with CML, the rate of progression of imatinib-treated patients was 3.3% [51]. A study carried out in Japan reported 7.5% (*n* = 16) of CML patients progressed to advanced phases [49]. Variation in WBC and platelet counts in our subjects and patients from other populations shows biological differences in AP-/BC-CML patients from different geographic regions that might be due to ethnic variations, the different genetic basis of CML progression in different ethnic groups and differences in the approach to clinical management of CML [45,49,50,51]. A higher frequency of CML progression in our patients can be attributed to the unavailability of all FDA-approved drugs, very few bone marrow transplantation centers and noncompliance of CML patients. It necessitated finding some early biomarkers of disease progression for our CML patients.

As there are no common molecular biomarkers available for early detection of CML progression [52], we subjected our advanced-phase CML patients to exome sequencing and compared them with CP-CML and healthy controls. We found that the ANKRD36 gene was exclusively mutated in all BC- and AP-CML patients but in none of the CP-CML patients and healthy controls. ANKRD36 is a novel gene that is still uncharacterized in humans. Nevertheless, the maximum expression of ANKRD36 is reported to be in myeloid cells of the bone marrow [53]. It is located on chromosome 2q11.2.

ANKRD36′s main function and exact role in CML or any other cancer are still unknown. However, various studies found an association between specific health conditions and ANKRD36. In type 2 diabetes mellitus patients (T2DM), ANKRD36 expression was found to be significantly upregulated as compared to normal controls [54]. CircANKRD36 (circular RNA transcribed by ANKRD36) level was positively correlated with glucose, glycosylated hemoglobin and IL-6. Furthermore, leucocytes expressed high levels of circANKRD36 in T2DM patients. Therefore, circANKRD36 may be used as a biomarker for screening chronic inflammation in patients with T2DM [54]. Another study showed an association between pneumonia pathogenesis and circANKRD36 [55]. Irritated MRC-5 cell injury by lipopolysaccharide (LPS) promoted the activation of the NF-κB signaling pathway by circANKRD36 and caused inflammation in MRC-5 cells. When circANKRD36 was silenced, the NF-κB pathway was inactivated, and this significantly increased the viability of LPS-aroused MRC-5 and decreased cell apoptosis [55]. Moreover, a similar study revealed the association between circANKRD36 and NF-κB pathway activation in H9c2 cells treated with LPS [56]. These studies show that ANKRD36 mutations can be categorized as “likely to be pathogenic”, and this gene may have a role in CML biology and progression.

Our protein biomodeling studies also indicate that ANKRD36 mutations reported by us fall under the category of “likely to be pathogenic” genetic alterations. ANKRD36 protein participates in diverse functions as transcriptional initiators, cell cycle regulators, cytoskeletal and ion transporters and signal transducers. Of clinical significance, natural variations in several ankyrin proteins have been previously reported to affect the specificity of protein interactions [57,58]. Mutation effect due to simultaneous “deletion of GC and insertion of TT” results in two amino acid changes: Ala to Cys (395) and Val to Phe (396). Both Val and Phe are hydrophobic, positionally interchangeable and resonate the same overall protein function because protein function is preserved due to retention of specific nucleotides in the DNA codon that encode amino acids with similar polarity or hydrophobicity substitution [59]. Nevertheless, A395C mutation has not been previously reported and might be of more importance, as rare mutations are more pathogenic than the frequent ones. The mutation location is on the surface exterior linking the two alpha helices and might alter the flexibility of the protein. This might hamper the potential interaction with other interacting proteins [48,57,58,59]. Possible predictions of functional annotation of partially characterized proteins and their functional domains surely need further validation.

We searched “The Cancer Genome Atlas (TCGA)” of the National Cancer Institute of the National Institute of Health (Bethesda, MD, USA) and “cBioPortal for Cancer Genomics” to find any leukemia-specific ANKRD36 mutations. Nevertheless, we could not find ANKRD36 mutations related to any type of leukemia. However, various studies have found a role of ANKRD36 in different cancers. A study analyzing the antitumor role of miR-144-5p in renal cell carcinoma (RCC) showed that the ANKRD36 gene is targeted by miR-144-5p [60]. In this study, poor survival was associated with high expression of miR-144-5p-regulated ANKRD36. Data from miRTarBase database of micro-RNAases shows that ANKRD36 is also regulated by miR-182, which is a miRNA expressed in the early stages of tumor growth [61]. A study showed that the silencing of miR-182 enhanced apoptosis. Moreover, a reduction in tumor growth was observed in vivo when anti-miR-182 treated cells were transplanted in immunodeficient mice. From these studies, it can be inferred that ANRD36 has a role in carcinogenesis and in the regulation of apoptosis. Moreover, it also indicates that silencing of ANKRD36 *miR-182* and *miR-144-5p* can suppress tumor growth and increase the apoptotic activity of the cancer cells. Thus, inhibition *of miR-182* and *miR-144-5p* might be important drug targets to find a new treatment for advanced phases of cancers where ANKRD36 has some role, including CML [61]. In another study, the mutational status of ANKRD36 genes was found to be correlated with proximal gastric cancer [62]. ANKRD36 has been reported to be coexpressing and interacting with other genes on locus 2q11.2, including ANKRD36C, ITPRIPL1, FAHD2B, FAM178B and CNNM3, which shows that ANKRD36 is involved in some important biological networks associated with cancers [63]. Studies have also found that ANKRD36 is upregulated by PIM1 inhibitors [64]. All these studies highlight the significance of ANKRD36 in important biological functions and its association with cancer, as well as showing that this gene is targetable and druggable if found mutated. As this gene has been found to have the highest expression in myeloid cells of the bone marrow, it may serve as a novel biomarker and drug target for CML patients with advanced phases of the disease [53]. Further studies are recommended for the biological characterization of this gene in humans and the identification of its possible role in CML progression and pathogenesis of other diseases.

In our studies, two out of three variants were not confirmed using Sanger sequencing. These variants may arise as a result of inevitable technical artifacts that are not uncommon in NGS-based studies and might be due to a number of reasons. Next-generation sequencing techniques generate low-interest variants in the form of genotype false positives. Biases in the library construction may lead to errors [65,66,67,68,69]. Moreover, we used NextSeq for WES, and this technology generates short reads. It is difficult to call genotypes at the end of short reads [70]. False positives in NGS data may also arise as a result of misalignment of sequencing reads to the reference sequence and inaccuracies or biases of the reference sequence compared to a specific local population [71]. Therefore, these factors should also be kept in mind during NGS-based investigations to avoid false-positive results.

## 5. Conclusions

We report mutations in a novel gene ANKRD36, which is associated with disease progression in CML and hence can serve as an important biomarker to identify CML patients at risk of disease progression. Our protein biomodeling studies show that these mutations change the structure of ANKRD36 protein, which might affect its biological functions. Although this gene is yet to be characterized in humans, various studies indicate its involvement in different biological functions and pathogenesis of diseases, including cancer. As this gene has been found to have maximum expression in bone marrow, specifically myeloid cells, it may have an important role in hematopoiesis and a therefore a potential role in hematopoietic diseases, specifically in CML progression. Accordingly, we recommend further studies to determine the exact biological functions of this gene, specifically its role in apoptosis and cancer carcinogenesis.

## Figures and Tables

**Table 1:** Statistics obtained before alignment of reads with the reference genome.

Statistics	ID 1	ID 2	ID 3	ID 4	ID 5
Total Number of Reads	70,508,170	75,173,754	75,622,396	71,328,320	76,940,162
Q30 (%)	96.6	97.0	96.8	96.9	97.1
Average Read Length (bp)	101.0	101.0	101.0	101.0	101.0
Total Yield (Mbp)	7121	7592	7637	7204	7770
Target Region (bp)	60,456,963	60,456,963	60,456,963	60,456,963	60,456,963
Average Depth (X)	117.7	125.5	126.3	119.11	128.5

Total yield = total number of reads x average read length; average depth is the throughput depth of the target regions (X) = total yield/target regions.

**Table 2:** Statistics obtained after alignment of reads with the reference genome.

Statistics	ID 1	ID 2	ID 3	ID 4	ID 5
Initial Mappable Reads	70,471,133	75,143,023	75,592,332	71,300,726	76,912,816
%Nonredundant Reads	88.1	86.0	86.9	86.3	87.1
%On-Target Reads	75.2	77.9	77.7	78.0	77.7
Depth of Target Region (X)	69.1	74.4	75.5	70.9	76.9
Coverage (% >10X)	97.0	97.3	97.3	96.9	97.1
Coverage (% >30X)	82.1	84.0	84.6	82.9	84.2

Initial mappable reads: number of reads mapped to human genome; %nonredundant reads = 100 × nonredundant reads/initial mappable reads; %on-target reads = 100 × on-target reads/nonredundant reads; on-target yield (bp) = the sum of the bases in the final alignment to the target regions; mean depth of target regions (X) = on-target yield/target regions; coverage statistics: the percentages of bases in target regions with a depth of coverage are mentioned.

## Data Availability

Data generated from next-generation sequencing have been submitted to NCBI and can be accessed at https://www.ncbi.nlm.nih.gov/sra/PRJNA734750 (SRA accession number PRJNA734750), accessed on 8 August 2021.
